# Embedded racism: Inequitable niche construction as a neglected evolutionary process affecting health

**DOI:** 10.1093/emph/eoad007

**Published:** 2023-04-21

**Authors:** Paula Ivey Henry, Meredith R Spence Beaulieu, Angelle Bradford, Joseph L Graves

**Affiliations:** Department of Social and Behavioral Sciences, T. H. Chan School of Public Health, Harvard University, Boston, MA, USA; Triangle Center for Evolutionary Medicine (TriCEM), Duke University, Durham, NC, USA; Department of Physiology, Tulane University School of Medicine, New Orleans, LA, USA; Department of Biology, North Carolina A&T State University, Greensboro, NC, USA

**Keywords:** racism, health disparities, niche construction, evolutionary mismatch

## Abstract

Racial health disparities are a pervasive feature of modern experience and structural racism is increasingly recognized as a public health crisis. Yet evolutionary medicine has not adequately addressed the racialization of health and disease, particularly the systematic embedding of social biases in biological processes leading to disparate health outcomes delineated by socially defined race. In contrast to the sheer dominance of medical publications which still assume genetic ‘race’ and omit mention of its social construction, we present an alternative biological framework of racialized health. We explore the unifying evolutionary-ecological principle of niche construction as it offers critical insights on internal and external biological and behavioral feedback processes environments at every level of the organization. We Integrate insights of niche construction theory in the context of human evolutionary and social history and phenotype-genotype modification, exposing the extent to which racism is an evolutionary mismatch underlying inequitable disparities in disease. We then apply ecological models of niche exclusion and exploitation to institutional and interpersonal racial constructions of population and individual health and demonstrate how discriminatory processes of health and harm apply to evolutionarily relevant disease classes and life-history processes in which socially defined race is poorly understood and evaluated. Ultimately, we call for evolutionary and biomedical scholars to recognize the salience of racism as a pathogenic process biasing health outcomes studied across disciplines and to redress the neglect of focus on research and application related to this crucial issue.

Despite expanding recognition of the importance of evolutionary medicine in academic and medical settings—and increasingly in the public sphere—the field of evolutionary medicine has not adequately addressed why some groups of people are consistently sicker than others. Racialized, ethnocentric and other forms of social discrimination are prominent contributors to the physical and mental health burden of populations and a leading source of health disparity across chronic and acute health outcomes nationally and globally [1–4]. These implicit and explicit biases are prevalent in medical science and delivery [5, 6], and structural racism is recognized as a public health crisis [7, 8]. However, a quest for deeper understanding of mechanisms by which racial biases cause differential disease outcomes is insufficiently explored in biomedical and evolutionary medicine research.

A recent study found that less than 1% of articles in four major medical journals from 1990 to 2020 contained the word ‘racism’, and of those that did, 90% were commentaries as opposed to research articles [9]. Similarly, our review of evolutionary medicine textbooks published in the last decade identified only two texts that included the terms ‘racism’, ‘health disparities/inequalities’ or ‘discrimination’ in the index [10, 11], excluding the few evolutionary texts that were specifically about socially defined race and its relationship to health outcomes [12–15]. A search of articles in *Evolution, Medicine, and Public* Health, a leading evolutionary medicine journal, shows only 5 on health disparities (<2% of 268 articles archived on Web of Science; Topic Search=(‘health disparit*’ OR ‘health ineq*’) accessed on 8 March 2023), and of these, none mention racism (Topic Search=((‘health disparit*’ OR ‘health ineq*’) AND ‘racism’) accessed on 8 March 2023). Racial health disparities, like other biological outcomes, cannot be isolated from evolutionary processes, and closely examining the determinant sources and mechanisms involved from an evolutionary medical lens can provide critical scientific insights. Evolutionary medicine, therefore, requires an integrated framework that broadens our view of health causation and recognizes the salience of racism and related forms of social discrimination to disease processes.

Evidence that humans do not have biological races has been written extensively [12, 14, 16–19]. Geographically based biological variation does exist within our species, and some of that variation is relevant to potential differences in disease prevalence between human groups around the world. In addition to this biological variation, some cultures have devised socially defined races, resulting in artificial (but socially realized) ‘races’ delineated via a shifting hodge-podge of criteria including physical and cultural traits, such as skin color, religion and language [14, 15, 20, 21]. Similar socially defined classifications facilitate other forms of subgroup discrimination (e.g. caste, colorism, ethnicity) [22, 23], and share origins in stratified sociopolitical hierarchies [11, 19, 20]. These differences in biological ‘race’ and socially defined race are critically important, yet poorly understood and applied in biomedical fields. A systematic review of medical publications in PubMed using self-reported race as a variable found no mention of race as a social construct [24]. This default genetic interpretation is at odds with established evidence in biological anthropology and public health science, and result in medical harm.

Despite the lack of biological races in humans, analyses of socially defined race remain relevant to evolutionary-based views of disease [20]. There is an adequate theoretical basis for examining racial health disparities within evolutionary medicine, as evidenced by works of JLG and others [25–28], in addition to substantial supporting theory and research in biological and biosocial anthropology [12, 19, 29–31]. Burgeoning epidemiological evidence has also generated new pathways to explore this challenge [1, 32–34], but evolutionary perspectives have not been adequately applied to social and discriminatory sources of health and disease, including racial inequities. The broad, systemic impact of institutional and interpersonal racism on biological processes [1] underscores the urgency to advance an evolutionary-ecological understanding of disease disparities beyond conventional evolutionary and biomedical approaches [35, 36].

What follows is a presentation of the unifying evolutionary-ecological framework of niche construction theory to examine processes by which racism and other discriminatory norms become embedded (i.e. constructed) in biology and disease. We first address racism as a social evolutionary mismatch, and apply the predictive processes of niche construction to investigate interacting ecological processes of biology, behavior and disease in racial health disparities. Niche construction unifies crucial processes of intrinsic and emergent biological-environmental feedback systems of disease variation over time, implicit in evolutionary queries of health. We make explicit the niche processes resulting in racial disparities and explore the relevance of competitive ecological models of niche exclusion and exploitation in these processes. Implications of racism and subordination on evolutionary domains of disease and life history are presented as evidence of novel divergent pathways biasing disease occurrence and progression. We close by discussing the urgent need for active evolutionary and biomedical examination and *re*construction of discriminatory and racist systems and effects to address these preventable harms.

## RACIALIZED HEALTH AND DISEASE: THE EVOLUTIONARY MISMATCH OF AN INEQUITABLE NICHE

Humans live in environments that differ drastically from those in which our shared African ancestors evolved [14, 19, 37, 38]. This discordance between evolved phenotypes and novel ecologies we now experience is of central interest to evolutionary medicine, as biological and behavioral mismatches can result in poor health and disease [11, 36, 39, 40]. Similar to adverse alterations in diet, activity, pathogens and microbiota, racism and racially stratified hierarchies are a consequential evolutionary mismatch for human health [10, 19, 30, 41]. From a medical view, evolutionary mismatch is typically concerned with biological traits, but expansion to examine mismatches of social traits is necessary, as culture and behavior are likewise rooted in evolutionary processes of our species’ history, with the potential consequences for wellbeing [31, 36, 42]. For example, novel social environments are embedded in food choices, activity, urban life and antibiotic use [11, 38, 41, 43–45]. Evolutionary mismatches challenge us to understand environmental processes underlying disease. These occur in biological and behavioral feedbacks from the allelic and epigenetic level to higher orders of organization (e.g. energetic, ontogenetic, cultural) in synergistic, rather than independent, ways to alter health [30, 31, 46, 47]. Health contexts are rarely uniform across large populations, and it is well established that racism segregates psychophysiological experiences for discriminated groups, resulting in damaging stress and trauma across the lifespan and generations [1, 14, 48].

Gene-centric views of natural selection and normative trait-based biology still dominate evolutionary thinking in biomedicine, limiting environmental analyses of disease processes and facilitating ready misuse of race in medical research and practice, including study design, clinical decision-making and now algorithms based in artificial intelligence [21, 24, 35, 36, 47, 49]. Examination of racism and its covariates fills critical shortfalls in evolutionary and biomedical research, as disease variation is fundamentally contingent on internal and external ecological contexts of health. Moreover, the causal weight of social and environmental inequity on disease has increased dramatically over the last century [2, 50, 51].

### Parameters of a racialized mismatch

A challenge of mismatch research biasing the way we think about race and health is that, to the extent they are considered, causal hypotheses of the human evolutionary niche are often not aligned to current science, and instead skew toward outdated theories of the human past [14, 17, 19, 49]. Research designs derived from biased and inaccurate normality frames influence how we understand what is mismatched about current, historic and evolved conditions. Evidence characterizing Pleistocene environments and the adaptive complex of our biological and social evolution has sharpened over recent decades, shedding new light on *Homo* and human ecological experience over 2 million years as hunter-gatherers, and relatively minute time as farmers, herders and urban dwellers [38, 41, 46, 49, 52]. At least four mammalian orders display health and lifespan effects related to the level of social integration, support and status [42], indicating that forms of social health determinants have deep evolutionary origins, where adverse social dynamics can affect survival. In *Homo* evolution, this vulnerability is central to its solution, as social sensitivity becomes specialized in a suite of biobehavioral and life-history adaptations, including cooperative food acquisition and distribution, reproductive dependency and extended development [46, 53–58].

In contrast, popular science is attracted to antagonistic notions of ‘human nature’, with selective attention to bellicose dominance hierarchies among some primates (e.g. chimpanzees, *Pan troglodytes*), violent conflicts between high-density horticulturalist-foragers (e.g. Yanomami), and evolutionarily recent bioarchaeological evidence of warfare, reflective of our own stratified cultural values (e.g. violence toleration, status-seeking) [37, 49]. While conflict is inherent in social experience, our species’ legacy of social tolerance between non-kin and cooperative resolution is evident among hunter-gatherer societies, characterized by quasi-egalitarian forms of exchange [46, 52], shared parenting [59], high residency of non-kin [60], flexible forms of leadership attained by cooperation and consent [61] and limited violence [37, 46, 55]. This evolutionary biological and social niche history underscores that racism has no *a priori* basis in age, strength, skill, kinship or evolutionary social structures [37, 62].

Race is a novel construct not based on universals, such as matrilineal or patrilineal descent. If suggestive of kin preference (e.g. in-group out-group discrimination), racial categories miss the genetic mark, socially and scientifically [12–14, 19]. Instead, racism recruits and distorts cognitive biases to align with dominance goals of exclusion rather than biological affiliation, as noted by the essential linkage of socially defined race to status hierarchies not determined by kinship [63]. Racial constructs are often absent cross-culturally (or recently introduced) [64], and their developmental presence is dependent on social priming [64–66]. Therefore, institutional social stratification, subordination and exclusion by socially defined race represent novel, harmful evolutionary mismatches of high-density populations, where resources are concentrated and controlled by status enforcement. This is altered from the demands of our more cooperative, tolerant, egalitarian evolutionary past, resulting in discriminatory disease and health consequences today [11, 19, 31, 36, 38, 47, 49, 58].

## NICHE CONSTRUCTION: PROCESSES OF HEALTH AND HARM IN RACIALLY CONSTRUCTED ENVIRONMENTS

Biomedicine still uses ‘race’ as a biological predictor of disease, not only in expectation of genetic insights on outcomes (with scarce query of error and bias [9, 45]), but as representations of an implicit set of correlated ‘lifestyle’ risks or ‘underinvestments’ in health [67, 68]. Yet standard analyses of disease differences fall far short of comparing the paucity of resource and health investments that discriminated communities receive [1, 3, 69]. To understand the evolutionary mismatch of health racialization and inequity, relevant theory is required to model the biological processes of social experience [70]. It is helpful for health research to see that experience in a new way. Biology, like evolution, is inherently an ecological process. But biomedicine lacks an advanced ecological view of the recursive phenotypic and genotypic feedbacks of environment and biology in the construction of disease [71]. Niche construction theory examines the role of intra- and inter-individual environments as integral components of these processes (e.g. [11, 37, 47, 72, 73]).

Niche construction is a productive theoretical framework for investigating the mismatched nature of health disparities, as it applies evolutionary and ecological principles to model interactive processes between environmental, phenotypic and genotypic systems across species [47, 74], including humans (e.g. [11, 19, 38, 49]). A product of the extended evolutionary synthesis, niche construction describes diverse pathways of phenotypic and evolutionary change not accounted for by random mutation or gene-directed inheritance alone, but by phenotype-environment feedbacks (e.g. individual, social, physical) and processes of biological embedding (e.g. sensorimotor, cognitive, homeostatic, developmental, disease) [71, 75–79]; i.e. genes as followers, not leaders in the process [77]. It explains evolutionary change generated by ecological, epigenetic and cultural inheritance; genetic accommodation and assimilation; intra-genotypic variation and biological canalization and plasticity, whereby the shifted phenotype becomes embedded in the genome, influencing the coevolution of traits, and direction and strength of selective processes [80–85]. As such, it counters context-free analyses of genetic and disease outcomes, and queries inherent environmental processes in biology, directing attention to individual and population agency in niches they inhabit and modify.

Critically, niche construction follows ecological advances on systems of environmental feedback and modification in other species (e.g. microbiomes, infectious vectors and agents, comparative species habitats and adaptations), traversing levels of biological and behavioral organization to focus on causal variation in sources and processes, not only products, of evolution and health [47, 72, 74, 78]. It broadens the theoretical and empirical horizons of health causation, facilitating collaborations with social, environmental and other biological sciences (e.g. [47, 52, 72, 77]). Niche construction is well-documented across species from cyanobacteria to humans, yet still remains a ‘neglected process in evolution’ [47, 79].

Regarding humans, niche construction models extend from progress in biological and biosocial anthropology on understanding internal and external ecologies of *Homo* evolution, diversity, and health, and have been productive to investigate coevolutionary processes of biological and cultural evolution [19, 37, 49, 52, 58]. These processes inform the impact of racism on health and disease, as physiological, social, and behavioral actions and responses, directly and indirectly, influence niche experience, altering subsequent conditions that organisms encounter and must adapt to. For example, in contrast to the ill-defined ‘ghost variable’ of race common in biomedicine [86], residential and employment segregation and discrimination experience more proximately and consistently predict health disparities [1–3, 30, 45, 87, 88]. These, in turn, shape future interactions in the racially defined niche. Niche construction processes typically reduce variability to create a more stable and predictable phenotypic experience (e.g. homeostasis, housing, caregiving, status protection), but variability can also be favored (e.g. genetic ‘bet-hedging’, phenotypic and developmental plasticity) [77, 89]. For example, while racism can severely narrow health options, these constraints can result in a diverse set of responses to evade its ill effects [90, 91].

Critical for analyses of evolutionary mismatch, niche construction bridges processes of highly stable evolved traits (e.g. morphogenesis, homeostasis) with environmental tradeoffs and phenotypic responses observed at shorter time frames. Thus, it can characterize adaptation with and without inferring trait selection at evolutionary scale (e.g. ontogenetic, metabolic, cultural and technological processes) [11, 41, 47, 66, 92, 93]. In contrast to narrow conceptualizations of gene selection, novel or altered system feedbacks between relatively stable traits and adaptive processes under fast-changing environments, including cancers, chronic and infectious disease, and culture, can negate or reverse the favorability of a particular allelic profile with each generation [19, 72, 74, 94]. This is key given the very recent timescale for the development of structured hierarchies and construction of racist systems [12, 14, 37, 43].

### Ecological models of competitive exclusion

The demonstrated impact of social discrimination on health and disease [1–3, 8, 20, 27] highlights a crucial factor in niche construction: individuals are not the only active constructors of their environment, but share environments actively constructed by others. Stratified hierarchies, especially racial hierarchies, generate harmful survival and reproductive conditions for subordinates. Politico-economic hierarchies capitalize on the evolved benefits of social dependency for status holders, but heighten health tradeoffs for designated groups instead [25, 31, 38, 41, 49]. Ethnoracialized systems partition and fortify inequitable resource access using tools of threat and discrimination [8, 90, 95]. This steepening stratification intensifies competitive norms and resource and reproductive demands, while weakening cooperation (a threat to status) at its base, with profound consequences for wellness and disease [4, 11, 19, 63].

Such actions reflect the ecological process of niche exclusion, whereby resource holders (attained by luck, ‘first-come’ status, inheritance or force) secure a dominance advantage by displacing and subordinating others, reducing competition over optimal or favored resources. Niche exclusion requires means to secure, sequester and defend high-value resource patches, while diminishing the capacity of others to safely obtain them [96]. Racism facilitates this subordination, as efficiency and effectiveness feedbacks from discriminatory cultural innovations (e.g. ready social cues, biased institutions and technologies) in the segregated niche benefit dominant groups, reifying constructions of ‘race’ as barriers to resource acquisition. These processes are informed by competitive and predatory exclusion feedbacks observed in other species, as diverse organisms face resource-sharing and harm-avoidance challenges of conflict, tolerance and cooperation/symbiosis determining future biological and behavioral tradeoffs. For example, active niche exclusion is observed in territorial behaviors, and inter-trophic displacement effects of predation on prey behavior, demonstrating how threat and insecurity generate an ‘ecology of fear’, shifting locality, resource acquisition and reproduction in the absence of direct assault [52, 96, 97].

This is relevant to health variation in humans, as discrimination structures create and enforce multiple constraints on individual and shared resource access, actively compromising subordinate capacity to compete (e.g. residential, education, employment segregation) [4, 95, 98]. Racism produces a persistent ‘ecology of fear’ with elaborated forms of violence, threat and deceit. Social race becomes a biological reality in these experiences, reflected in the predictive weight of race/ethnicity categories on discrimination measures, the extent of active resource sequestration, and the impacts of each on health and disease [1, 23, 99]. Overt (e.g. violence, redlining) and latent (e.g. norms, stereotypes, vicarious) discrimination have measurable effects on disease progression via a cascade of responsive biological processes, representing an evolutionary mismatch to novel social stressors [30, 91, 97]. For example, psychophysiological processes associated with social discrimination variously include high blood pressure, cardiovascular and metabolic disease, hypothalamic-pituitary-adrenal (HPA) axis responsivity, C-reactive protein, anxiety, depression and other neurophysiological responses [4, 34, 90, 100, 101]. Rather than evidence of group genomic vulnerabilities, their occurrence reflects active niche feedbacks of deprivation and hazard from social and resource exclusion itself [45, 91]. In contrast, high-status exemption from racist threats constructs protective, expansionist feedbacks (e.g. confidence, strategic risk-taking) that enhance economic and reproductive security and opportunities [96, 97].

Intensive human status hierarchies do more than exclude designated others from individual and cooperative benefits: they facilitate energy capture from them. Exploitative economic systems put subordination to use, structuring niche expansions that reduce high-status time and energy expenditures, compounding resource gains. Co-option of subgroup time, labor and resource captures (e.g. resource extraction, colonization, slavery, exploitative work, predatory lending, incarcerated labor) requires enhanced enforcement and opportunity constraints on subjugates, sharply prejudicing evolutionary, ecological and health tradeoffs for discriminated groups (e.g. [11, 14, 98]). Racialized exploitation also has destructive effects on lived environments, not only given prior unfavorable conditions of niche exclusion (e.g. less productive land, poor infrastructure) [95, 96], but because the externality costs of niche expansion and exploitation (e.g. habitat loss, pollution, health costs) are systematically displaced to subordinated space [80, 81, 102].

A niche ecological perspective on racism and health exposes interpersonal and institutional agency in the construction of biological and habitat feedbacks of social and resource exclusion, capture and threat. Rather than a predictable side-effect of ‘human nature’, racism and systems that uphold it represent a distortion of protective social biases, and a stark break from our evolved biological interdependence of cooperative life history, economic exchange and conflict-solving. This mismatch is characterized by steeply stratified social systems designed to secure and expand the resource and reproductive favorability via status, power and social bias, garnering social, economic and health assets while redirecting and enforcing health-punitive costs on segregated others [8, 14, 19]. As such, predicted disease consequences of discriminatory exclusion and threat likely reflect the heavy survival and reproductive toll of social ostracism in human evolutionary history.

## RACIALLY CONSTRUCTED HEALTH OUTCOMES OF THE EVOLUTIONARY MISMATCHED NICHE

This niche-construction view of racialized health is necessarily incomplete but can sharpen research and clinical practice by demonstrating the relevance of evolutionary-ecological principles to health disparities. Ecologies of racism permeate a diverse set of disease processes, resulting in divergent health processes, treatments and outcomes. These patterns reflect biological impacts of niche exclusion and threat, and account for more variation in disease than conventionally considered in evolutionary medical research. Niche construction principles can guide a more systematic examination of divergent outcomes across chronic and complex diseases, infectious diseases, and diseases of homeostasis.

**Chronic and complex diseases**, such as heart disease, cancers, respiratory disease and cerebrovascular disease, are the leading biological causes of death in the USA [83]. Genetic variation contributes to these diseases, but not in a way that can account for the well-known disparities observed between socially defined racial groups. For example, in coronary artery disease (CAD), genome-wide association studies have demonstrated that heritability (*h*^2^ = 0.40–0.60) of this condition is approximately the same for African Americans, Hispanics and European Americans. However, the majority of studies have been conducted on persons of European descent. These studies identified between 208 and 321 single-nucleotide polymorphisms (SNPs) accounting for only 15% of CAD heritability. Furthermore, the locus with the highest known effect in European-descended populations (9p21) was not found at all in African-descended populations [82]. Meanwhile, the Millions Veterans study of ~250,000 persons found 95 novel SNPs not reported in European populations associated with CAD. These results underscore the fact that heritability estimates are highly dependent on the subjects included in the study and reflect unmeasured environments embedded in both genotype and phenotype.

Conversely, well-known environmental and behavioral risk factors for heart disease include poor diet, sedentarism, ambient air and noise pollution, sleep deprivation and psychosocial stress, each a component of mismatched niche construction processes. Racism, too, presents a chronic threat that can become embedded in disease experience. There is strong evidence that psychosocial stress resulting from structural racism differently predisposes African Americans to heart disease [84], with differential levels of methylation in promoter regions associated with heart muscle failure by socially defined race and socioeconomic status [85]. Similar effects can be seen for lung cancer. Environmental risk factors for lung cancer include cigarette smoking, as well as exposure to various airborne pollutants, excessive alcohol consumption and occupational exposure [103]. In the USA, socially defined race plays a major role in one’s probability of being exposed to excessive air pollution [104], illustrating how segregated niches are increasingly degraded. Historical and ongoing construction of systemic racism presents an environmental, psychosocial and physiological mismatched niche, consistent with long-standing disparity in heart disease [105] and lung cancer by socially defined race.

A potent construct of niche exclusion with chronic health effects is ‘redlining’, a term to describe the 1930s US federal mortgage policies that denied lending to those in predominantly African American, foreign-born or low-income residential areas. Redlining is associated with higher environmental and social health risks, and segregated forms of medical care, with a majority of African Americans born in areas with hospitals ranked lower in quality [106]. Those living in previously redlined neighborhoods received more late-stage cancer diagnoses for lung, colorectal, cervical and breast cancer compared to those in predominantly European American areas independent of race/ethnicity, age or income [107].

**Infectious diseases** are similarly associated with discrimination and exploitation and are expanded and perpetuated by new ecologies of racial inequity. A recent study of SARS-CoV-2 risk in New York City demonstrated that severity was predicted by the contours of formerly redlined neighborhoods [108]. Tuberculosis and other respiratory diseases are consistently associated with residential segregation and incarceration in the USA [109]. Infectious disease distributions in Indigenous communities are increasingly structured by racist exclusion and threats [110, 111]. For example, American Indians and Native Alaskans are over 3.7 times more likely to experience plumbing poverty—the lack of complete indoor plumbing—heightening infectious disease exposures [112]. Hepatitis was identified in 87% of South American Indigenous groups [113], and the prevalence of H1N1 in First Nations regions reached more than three times that of Canada overall during its spread [114]. Indigenous Africans also experience widespread socio-political exclusion, facilitating accelerated land displacement and labor exploitation, accompanied by risks of malaria, respiratory and gastrointestinal disease, hepatitis and sexually transmitted diseases that differ substantially from the past [115].

Racism also presents risks to somatic construction processes that stabilize health. Direct and indirect discrimination encounters have notable impacts on physiology and neuropsychological functioning, resulting in **diseases of homeostasis**. Contrary to medical hypotheses of racially distinct traits, variation in homeostatic feedbacks reflect active efforts to manage discrimination and threat, evidenced in physiological and psychobehavioral responsivity (and lack thereof) to chronic and unpredictable racist threats [91, 101, 116, 117]. Meta-analyses demonstrate increases in blood pressure, body mass index, cardiovascular disease and mortality in association with vicarious and direct experiences of racism [1, 116, 118, 119]. Alterations in stress reactivity via the HPA axis, C-reactive protein and immune system and autonomic nervous systems are regularly associated with discrimination encounters [90, 100], as are a suite of adverse mental health outcomes among children and adults [4, 48, 87, 120]. Racism degrades neuropsychological functioning and defense (e.g. cooperative/competitive effort, harm avoidance), but is systematically overlooked by clinical psychology [87]. The prevalence and embedding of racism underscore the challenge of disentangling the allostatic effects of exogenous disruption on homeostatic measures given their consistent correlation with social and resource segregation and stress [21, 121].

## PASSING HEALTH DISPARITIES TO FUTURE GENERATIONS: THE RACIALIZATION OF LIFE-HISTORY PROCESSES

An extension of racism’s impact on health and disease is its profound effects on survival and reproduction [14, 20]. The tethered nature of life history processes to niche conditions extends the pathogenic consequences of inequitable assets and risks across the lifespan and generations via biological and environmental niche construction processes (e.g. genetic and phenotypic variability and plasticity, epigenetic modifications, parental and niche inheritance) [10, 66, 91, 122]. Environmental ‘harshness’, variably measured, is the most consistent predictor of life history differences across studies [123], and local energy balance and risks can generate disparities within populations or in those inhabiting similar ecologies (e.g. [57, 69]). Meta-analyses demonstrate that racial discrimination is directly associated with adverse pregnancy outcomes [124], and consistent racial/ethnic risks on perinatal outcomes occur across primarily Western high- and middle-income countries [125]. These effects spillover to compound discriminatory harms in childhood [48, 66]. Following ground-breaking evolutionary work on epigenetic processes in racialized environments (e.g. [11, 25–27, 30, 91]), modern life-history comparisons require measures of the extent to which demographic and health patterns are constructed by discriminatory forms of reproductive exclusion and suppression [10, 43, 91]. Racism has a substantial impact on developmental, reproductive and health tradeoffs by compounding disparate resource and safety costs on already high allocation demands [4, 97, 98].

Meta-analyses show that developmental experience of discrimination adversely impacts child and adolescent asthma, cortisol levels, obesity, injury, mental health, dysregulation and substance use, which can alter health and development progress across the lifespan [48, 120]. A study of over 11,000 African American adolescents found that later pubertal stage by age was associated with greater discrimination experienced in the past year, controlling for other measures of adversity [126]. Social and environmental exclusion is also predictive of toxic exposures that can harm reproductive development [81]. Higher prepubertal traffic exposure is associated with an average 2–9 month increase in pubertal stage by age among US girls, with African American girls living 33% closer to heavy traffic than European American peers and demonstrating earlier pubertal progress at 9.9 v. 10.6 years [127]. US Akwesasne Mohawk girls’ exposure to estrogenic polychlorinated biphenyls was associated with earlier menarche, and a higher risk of autoimmune disorders [128].

Maternal discrimination is independently associated with adverse maternal and offspring outcomes, including higher pregnancy-related complications, preterm birth and infant mortality [88, 124]. Racialized risks directly skew life-history patterns, with effects predicted by residential, economic and education segregation, poor healthcare quality, legal/civil rights discrimination, police violence and mass incarceration [129–132]. Even acute indirect threats can rapidly alter reproduction. For example, a 2008 Ohio immigration raid that arrested nearly 400 predominantly Hispanic/Latino workers was associated with lower birth weights in these populations, but not European Americans, in the state in the 37 weeks that followed [133]. Racism, moreover, has adverse effects on mate access and relationships [134, 135].

Among Indigenous peoples globally and in high-income countries, persistent ethnoracialized niches of displacement, neglect and exploitation compound developmental and reproductive harms [115, 136]. Even as first births shift downward in age with Westernized exposures, accelerations of social and environmental exclusion and degradation depress fertility and survivorship in many groups, even below replacement levels [137, 138]. Thayer and colleagues [27] reported that Māori women, who experience the highest levels of ethnic discrimination among New Zealanders, had shorter gestation lengths after controlling for other socioeconomic and health factors, and reported associated antenatal instances of ethnically motivated physical attacks, unfair treatment at work, in banking and the criminal justice system. Echoing the toxic environmental risks of racialized segregation in the USA [81]. Indigenous people globally are disproportionately exposed to polluted water, land and air, affecting reproductive health, infant survivorship and development [139].

## RECONSTRUCTING THE NICHE: THE ROLE OF EVOLUTIONARY MEDICINE IN MOVING FORWARD

Striking global health disparities along social rather than biological lines demonstrate how consequential racism is as a tool for social, resource and reproductive exclusion [43, 118], segregating health experience across lifespans and disease systems [140]. Public health has well-demonstrated systematic harms of discrimination across health, and racism in diverse forms looms large in adverse health consequences. Clarity on temporal and spatial processes of phenotypic, genetic and disease modification is critical to dismantle racialized assumptions and actions that structure biomedicine, including biological stereotypes of human differences [9, 21, 45, 67]. Examination of racial health disparities through an evolutionary medicine lens highlights that divergent disease patterns are largely due to mismatched cultural constructions of social status, exclusion and exploitation enforced by discriminatory norms, threats and violence.

The scientific challenge to explain and redress racism’s harms is gaining attention, but there is scant empirical progress in biomedical operationalizations of ‘race’ in research or practice (e.g. [24]). Racist health mis/disinformation is increasingly aggressive and global [51], and meta-analyses of interventions designed to weaken prejudice find the majority of efforts were ‘light-touch’ with low impact [141]. Our efforts should not be so. That race is not biological and racism is a recent invention of our species offers little defense for inertia, as its pathological health consequences are rooted in the same mismatch processes that we routinely study in evolutionary medicine. Widespread medical misinterpretation of racial/ethnic constructs and genetic relationships must be addressed by the health discipline whose explicit mission is the application of evolutionary principles and an understanding of evolutionary history to disease processes (Box 1).

Racialized niches make it difficult to interpret the degree of plasticity and trait variation meaningful for population genotype × phenotype comparisons of disease. Divergent constructions of health and wellbeing constrain our ability to assess the range of underlying response variation across an environmentally comparative range, and measures of that experience are few. In the absence of a sustained substitution test of divergent niches, genetic influences cannot be independently differentiated from racialized effects [14, 21, 36, 142]. Nor can discriminatory experience be conceptually or analytically reduced to socioeconomic, allostatic or environmental harshness [1, 3, 67]. Unlike forms of scarcity and social conflict in our evolutionary past, racism presents a targeted, chronically hostile mismatch outside evolved biological and social reaction norms. In the absence of substantial niche *re*construction, contemporary ecologies of racism will perpetuate the recursive processes of past inequity, exploitation and trauma into present-day niches of threat and harm [10, 91].

Niche construction describes a set of ubiquitous evolutionary-ecological processes with critical research and clinical implications for discriminatory drivers of health. While evolutionary medical approaches shift the ‘health-deficit’ paradigm toward investigations of tradeoffs and mismatched conditions, only limited analyses explore how racially structured societies entrench advantageous health and life-history feedbacks of dominance (e.g. reducing competition, maximizing resource flow), while compounding disease and life-history costs on those subordinated (e.g. economic and reproductive exclusion, biological and psychosocial threat, allostatic harm) [98, 99, 143]. Crucially, niche construction theory requires an agent’s view, and rather than render subordinates passive subjects of constructed harm, it necessitates the participation and experience of those excluded. This dynamic framework bridges siloed research and theory elsewhere [32, 68], and can strengthen the construction of the anti-racist niche by exposing underlying processes—and opportunities—neglected by biomedicine and other fields.

Closer examination of how disease processes are altered by racial/ethnic health experience will expand our contribution to evolutionary understandings of health variation across the human niche, and inform long-standing assumptions of the human ‘norm’ [19, 35, 144]. The seeming intractability of race as a predictor of health and disease requires both an undoing of its biological assumptions, as previously argued [12–14, 29, 144], and the cultural undoing of racism with its capacity for exclusion and control constructed by systems, structures and norms that segregate and enforce the divergent niche [2, 3, 20, 31, 67]. Integrating advances on these processes and effects in evolutionary medical discourse, and addressing our social responsibilities as scientists and practitioners, will advance our disciplinary relevance in the pressing global challenge of understanding health disparities and enable progress toward the construction of a more equitable niche.

**Box 1. *Re*constructing the racialized niche of health and disease in evolutionary medicine**

Niche construction underscores the role of *agency* in biological and behavioral feedbacks. This necessarily includes the persistent role of science and scientists in racism’s harms. Medical neglect of racism [5, 6, 9] and the wholesale misuse of ‘race’ as genetic type [14, 24] amid substantial public health evidence on racialized health demonstrate the tenacity of false, harmful narratives in science. Given that inaccurate evolutionary assumptions underlie racist constructions of human diversity, evolutionary medicine scholars have a vital responsibility to become informed—and subsequently inform science and society—on racism’s social and biological processes and effects on health and disease. And, as systemic producers (i.e. constructors) of these harms, to assert an ambitious role in its undoing.

An example of this kind of engaged scholarship is illustrated by the work of JLG and his colleague Dr. Andrea Deyrup (MD, PhD, Pathology Department, Duke University), who have:

- Given more than 50 lectures on racial misconceptions in medicine at leading medical departments in the USA and Canada over the last two years [145];
- Engaged in removing racial medicine concepts and introducing an evolutionary medicine perspective on health disparities in one of the world’s leading pathology textbooks [146]; and
- Published papers in leading medical journals addressing ongoing racial misconceptions in medical practice (e.g. [147]).

Leaders are needed to champion similar efforts across personal and professional contexts in academic, research, clinical, and public settings. This includes the training of physicians and other health practitioners, evolutionary scientists, and scientists in biological disciplines broadly.

Evolutionary medicine as a field requires systematic anti-racist changes in research, education, and practice. This can begin to be accomplished through the following actions:

- Play an active role—through research, clinical practice and public discourse—in deconstructing fallacious evolutionary and genetic interpretations of race and its ascribed associated physical and behavioral values (see [24]).
- Open and protect space for those most harmed by racism to lead the conversation as experts in social and health experience, and actions in response. Active communication strategies can magnify new and accurate characterizations of race and health [148].
- Implement implicit bias training that includes strategies for mitigating such biases in medical training and clinical settings [149]. Advocate for legislation that mandates effective educational interventions on implicit bias for health professionals [150], ideally with sustained training that goes beyond a single required offering.
- Recognize that scientists, practitioners and educators are influential niche constructors. Become informed and re-examine our own scientific activities (e.g. study, practice and education designs, curricula, processes, measures, treatments and outcomes) for interpersonal, social and environmental sources of discriminatory bias. Join and recruit others in anti-racist reforms in educational, research and clinical settings.
- Actively deconstruct interpersonal and institutional barriers to recruitment and retention of diverse colleagues, students, subjects and patients in academic, research and clinical settings.
- Prioritize study metrics and analyses sensitive to population variation rather than ‘normative’ typologies alone. Explore demographic, experiential and environmental data as parameters of values in medical publications, including through interdisciplinary collaborations [32]. Utilize available well-designed, valid tools (e.g. [34, 88, 116]) to add personal and structural discrimination measures to research designs.
- Build on existing models from medical contexts (e.g. [151]) to develop and disseminate curricula that give faculty the appropriate content and facilitation skillset required to effectively instruct students from diverse fields in issues of race, inequitable niche construction, and racialized health outcomes.

This is a limited set of actions. General and within-discipline guides on anti-racism reforms are steadily increasing, and collaborative opportunities are expanding. Evolutionary medicine scholars can and should be leaders in shifting the inaccurate and pervasive narratives of ‘race’ to an evolutionarily informed understanding of racism as the primary driver of racialized health disparities.
